# Infected Complex Odontoma Associated With a Dentigerous Cyst Presenting as a Cutaneous Fistula in the Mandibular Angle: Surgical Management Using Piezoelectric Surgery

**DOI:** 10.1155/crid/2059316

**Published:** 2026-10-12

**Authors:** Imen Raâdani

**Affiliations:** ^1^ Faculty of Dental Medicine of Monastir University of Monastir Monastir Tunisia um.rnu.tn

**Keywords:** case report, cutaneous fistula, dentigerous cyst, impacted tooth, mandibular neoplasms, odontoma, piezosurgery

## Abstract

Odontomas are the most common benign odontogenic hamartomatous tumors. Although typically asymptomatic, they can occasionally lead to severe infectious complications. This report describes a rare case of an infected complex odontoma manifesting as a cutaneous fistula, emphasizing the surgical challenges associated with its anatomical location. A 22‐year‐old male presented with a 1‐month history of a cutaneous fistula at the left mandibular angle and intraoral bone exposure. Radiographic evaluation (panoramic and CBCT) revealed a voluminous radiopaque mass at the mandibular angle, which had severely displaced the third molar toward the inferior border of the mandible. The proximity to the inferior alveolar nerve and the thinness of the remaining bone posed a significant risk of iatrogenic fracture. The patient underwent surgical enucleation of the lesion and extraction of the displaced molar. To mitigate the risk of mandibular fracture and nerve injury, piezoelectric surgery was utilized. The use of ultrasonic inserts allowed for a precise, selective osteotomy and a safe extraction, preserving the structural integrity of the weakened mandible. Histopathological analysis confirmed the diagnosis of a complex odontoma associated with dentigerous cyst. At the 6‐month follow‐up, the cutaneous fistula had completely resolved with excellent healing and no sign of recurrence. This case highlights that for large, infected odontogenic lesions, the synergy between 3D imaging and minimally invasive technologies like piezosurgery is essential to achieve successful clinical outcomes while minimizing surgical morbidity.

## 1. Introduction

Odontomas represent the most prevalent category of odontogenic tumors, accounting for approximately 22.6% according to recent epidemiological data [1]. Classified by the World Health Organization as benign hamartomatous tumors, they are composed of a disorganized mixture of dental tissues, including enamel, dentin, and cementum, along with pulp‐like connective tissue [2]. These lesions are traditionally categorized into two distinct morphological types: compound odontomas, which consist of small, tooth‐like structures (denticles), and complex odontomas, where the dental tissues are arranged in a chaotic, nonanatomical mass [2].

Although the exact etiology remains unclear, factors such as trauma during primary dentition, local infections, or genetic mutations have been suggested as potential triggers [3]. Most complex odontomas are discovered during the first two decades of life, often incidentally during routine dental radiographic screenings [4]. Despite their benign nature and slow growth pattern, these tumors can reach significant dimensions if left untreated. Large complex odontomas frequently cause clinical complications, including delayed tooth eruption, significant displacement of adjacent teeth, and expansion of the cortical bone [5].

Clinical symptoms are usually absent unless the lesion becomes infected or erodes through the oral mucosa. In rare cases, such as the one presented here, an infected complex odontoma can establish a communication between the oral cavity and the skin, leading to a cutaneous fistula [6]. This extraoral manifestation is a diagnostic challenge for clinicians, as it can mimic other pathologies like branchial cleft cysts or actinomycosis. Recent advancements in 3D imaging, particularly CBCT, have become indispensable in assessing the relationship between the tumor and vital anatomical structures, such as the inferior alveolar nerve or the mandibular border [4].

The purpose of this report is to detail a rare clinical case of an infected complex odontoma in a 22‐year‐old male, which manifested as a persistent cutaneous fistula and intraoral bone exposure, and to discuss the surgical management strategies required to achieve complete resolution and prevent recurrence.

## 2. Case Presentation

This case report has been prepared in accordance with the CARE guidelines to ensure transparent and comprehensive reporting.

### 2.1. Patient Information

A 22‐year‐old male patient with no significant past medical history was referred to the department of oral surgery for evaluation of a persistent cutaneous fistula located at the left mandibular angle. The lesion had been present for approximately 1 month and was associated with intermittent purulent discharge. The patient denied pain, fever, or any constitutional symptoms.

His medical history was otherwise unremarkable. He reported no previous facial trauma or oral surgical procedures and denied the use of chronic medications. No systemic diseases known to impair bone metabolism, wound healing, or increase susceptibility to infection were identified. The family and social histories were noncontributory.

Before referral to our department, the patient had been treated with oral amoxicillin/clavulanic acid (Augmentin) at a total dose of 3 g/day for 15 days; however, purulent drainage persisted (Table 1).

**Table 1:** Timeline.

Time	Clinical event
1 month before consultation	Appearance of a persistent cutaneous fistula at the left mandibular angle with intermittent purulent discharge.
Before referral	The patient received oral amoxicillin/clavulanic acid (Augmentin) at a total dose of 3 g/day for 15 days because of the persistent purulent drainage.
Initial consultation	Clinical examination revealed a cutaneous fistula and exposed bone in the left retromolar region.
Diagnostic assessment	Panoramic radiography followed by CBCT identified a large complex odontoma associated with an impacted mandibular third molar.
Surgical intervention	Piezoelectric excision of the odontoma, extraction of the impacted mandibular third molar, and debridement of the cutaneous fistulous tract.
Histopathology	Diagnosis of complex odontoma associated with a dentigerous cyst was confirmed.
1‐month follow‐up	Marked improvement of intraoral and extraoral lesions.
3‐month follow‐up	Complete resolution of symptoms with satisfactory soft tissue healing.
6‐month follow‐up	Complete mucosal healing, minimal cutaneous scar, and no clinical evidence of recurrence or neurosensory deficit.

### 2.2. Clinical Findings

Extraoral examination revealed a cutaneous fistula at the left mandibular angle surrounded by mild erythema, with purulent drainage upon palpation (Figure 1). No significant facial swelling or cervical lymphadenopathy was observed.

**Figure 1:**
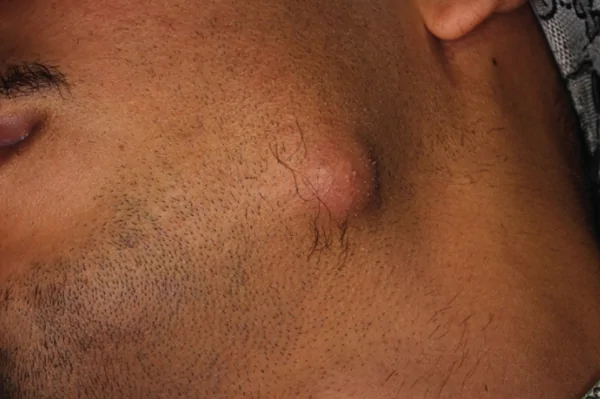
Extraoral clinical photograph showing a cutaneous fistula at the left mandibular angle.

Intraoral examination demonstrated an area of exposed necrotic bone in the left retromolar region associated with localized mucosal inflammation (Figure 2). The remaining oral mucosa appeared healthy, and no limitation in mouth opening or occlusal disturbance was noted. Neurosensory examination confirmed normal sensation within the distribution of the inferior alveolar nerve.

**Figure 2:**
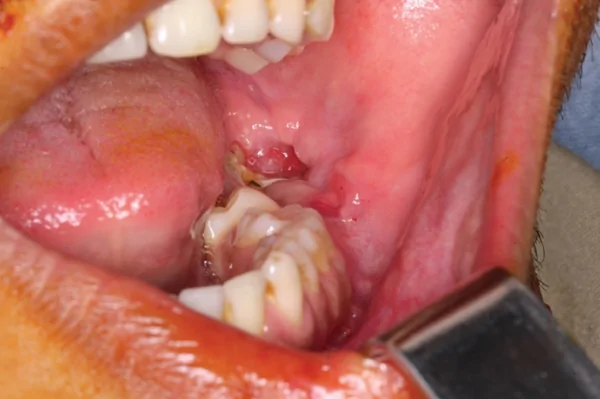
Intraoral clinical photograph showing exposed bone in the left retromolar region.

## 3. Diagnostic Assessment

Panoramic radiography demonstrated a large, well‐defined radiopaque lesion surrounded by a thin radiolucent halo occupying the left mandibular angle (Figure 3). The lesion displaced the impacted mandibular third molar toward the inferior border of the mandible.

**Figure 3:**
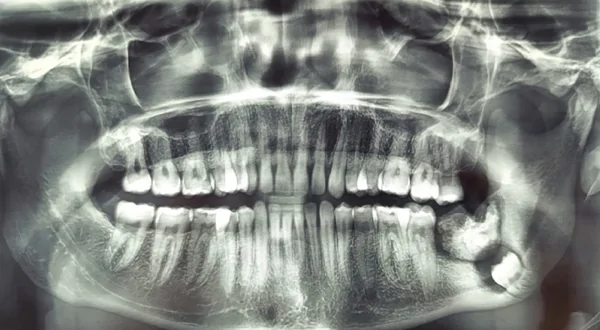
Panoramic radiograph showing a large, well‐defined radiopaque mass consistent with a complex odontoma, surrounded by a peripheral radiolucent halo at the left mandibular angle.

Pulp vitality testing of the adjacent teeth was performed and showed positive responses, with no clinical or radiographic evidence of pulpal necrosis or periapical pathology.

To further characterize the lesion and evaluate its relationship with adjacent anatomical structures, maxillofacial computed tomography (CT) was performed using a Philips scanner with helical millimetric acquisition, without intravenous iodinated contrast administration. The acquired images had a slice thickness of 0.86 mm. CT examination demonstrated a well‐defined hypodense mandibular lesion measuring 31 × 22 mm in maximum dimensions, involving the left mandibular angle and encompassing the impacted third molar. The lesion was associated with cortical bone lysis and infiltration of the adjacent soft tissues, as reported on the radiological examination. Its close relationship with the inferior alveolar canal was also noted.

The examination also demonstrated marked thinning of the mandibular cortex and the close relationship between the lesion and the inferior alveolar canal (Figure 4).

**Figure 4:**
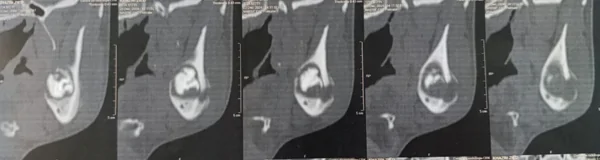
Coronal maxillofacial CT image showing a well‐defined hypodense mandibular lesion, measuring 31 × 22 mm in maximum dimensions and associated with an impacted third molar. Marked thinning of the mandibular cortical bone and a close relationship between the lesion and the inferior alveolar canal are also demonstrated. Slice thickness is 0.86 mm.

Based on the clinical and radiographic findings, the principal differential diagnoses included cemento‐ossifying fibroma, osteoma, calcifying epithelial odontogenic tumor, and chronic osteomyelitis. However, the combination of a well‐circumscribed radiopaque mass surrounded by a radiolucent rim, its association with an impacted tooth, and the subsequent histopathological findings supported the diagnosis of a complex odontoma associated with a dentigerous cyst.

### 3.1. Therapeutic Intervention

Several factors made surgical management particularly challenging. The considerable size of the lesion and its location within the mandibular angle significantly weakened the mandibular cortex, increasing the risk of intraoperative or postoperative pathological fracture. Furthermore, the displaced third molar was located in close proximity to the inferior alveolar canal, raising concerns regarding possible nerve injury during surgical removal. Finally, the presence of a chronic cutaneous fistula and exposed bone indicated persistent infection requiring complete elimination of infected tissues.

After obtaining informed consent, surgery was performed under local anesthesia. A full‐thickness vestibular mucoperiosteal flap was elevated to expose the lesion. Considering the proximity of the inferior alveolar nerve and the fragility of the mandibular angle, piezoelectric surgery was selected instead of conventional rotary instruments because the cavitation effect provided enhanced intraoperative visibility and contributed to a relatively bloodless surgical field.

Progressive ultrasonic osteotomy allowed conservative bone removal and careful enucleation of the calcified mass while preserving adjacent soft tissues. A Mectron piezoelectric device was used with the OP3 insert, in cortical cutting function with irrigation at Level 3. Following complete removal of the odontoma, the impacted mandibular third molar was extracted (Figures 5 and 6). The cutaneous fistulous tract and all inflammatory granulation tissue were thoroughly curetted and debrided before copious irrigation of the surgical field. The flap was repositioned and closed with interrupted resorbable sutures.

**Figure 5:**
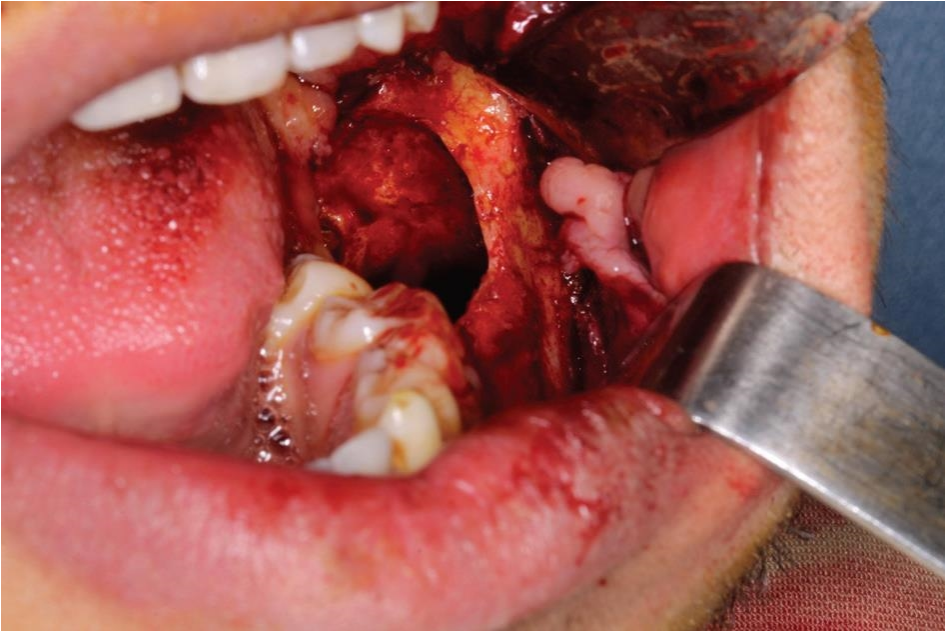
Intraoral clinical photograph showing the surgical site after excision of the complex odontoma and extraction of the associated impacted third molar.

**Figure 6:**
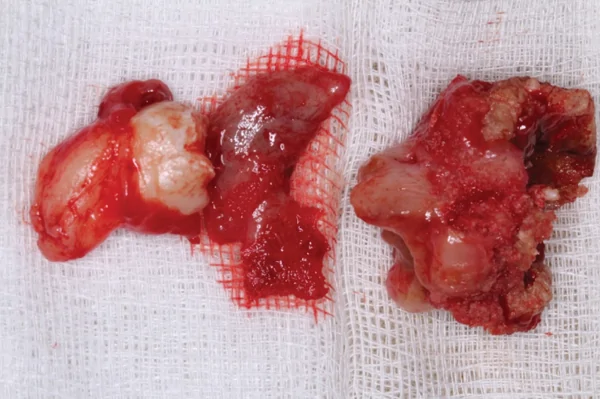
Surgical specimen showing the excised complex odontoma associated with a dentigerous cyst and the extracted impacted tooth.

The patient was prescribed Novaclav (amoxicillin 1 g + clavulanic acid 125 mg), orally, three times daily for 7 days, Panadol (paracetamol) 1 g orally, three times daily for 5 days, and chlorhexidine mouth rinses, initiated the day after surgery, for 10 days. Postoperative instructions also included meticulous oral hygiene and a soft diet to minimize mechanical stress on the weakened mandible during the initial healing period.

Histopathological examination of the mandibular specimen revealed a composite lesion consisting of a dentigerous cyst, an odontoma, and chronic inflammatory bone changes consistent with osteitis. In the upper portion of the section, a well‐demarcated and relatively homogeneous nodular fragment of acellular, intensely eosinophilic dental tissue was observed, characterized by the absence of cellular nuclei and consistent with odontomatous dental tissue. The middle portion of the section was composed of a thick band of dense fibrous connective tissue forming the cyst wall. Focally, the inner surface of this fibrous wall was lined by a thin, nonkeratinized stratified squamous epithelium corresponding to the cystic lining. The lower portion showed marked pathological bone remodeling, with irregular and eroded necrotic bony trabeculae exhibiting empty osteocyte lacunae. The intertrabecular spaces were extensively occupied by a dense polymorphous inflammatory infiltrate and granulation tissue, characterized by numerous inflammatory cells, supporting the diagnosis of chronic osteitis. (Figure 7).

**Figure 7:**
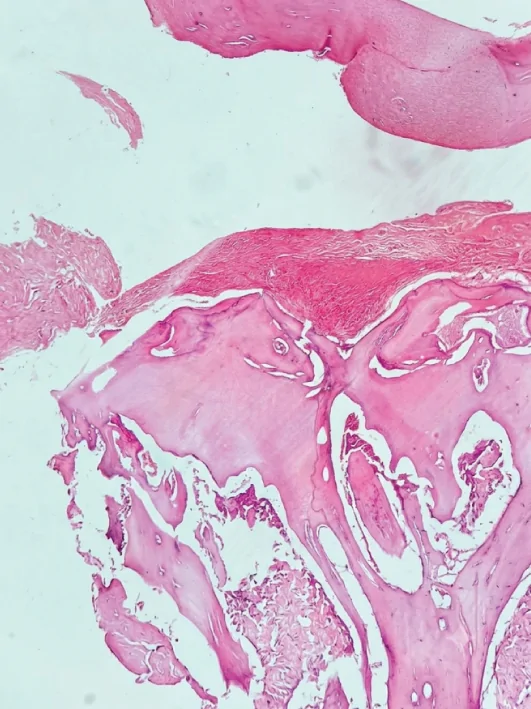
Histopathological examination of the mandibular specimen showing a complex odontoma associated with a dentigerous cyst and chronic osteitis (hematoxylin and eosin [H&E] staining, ×100 magnification). The upper portion shows a well‐demarcated nodule of acellular eosinophilic dental tissue consistent with odontomatous tissue. The middle portion shows the dense fibrous connective tissue forming the cyst wall. The lower portion demonstrates irregular necrotic bony trabeculae consistent with chronic osteitis.

### 3.2. Follow‐Up and Outcomes

The postoperative course was uneventful, with no evidence of infection, mandibular fracture, or neurosensory disturbance.

The patient complied with postoperative instructions and attended all scheduled follow‐up visits.

At the 1‐month follow‐up visit, substantial improvement of both the intraoral wound and the cutaneous fistula was observed, with complete cessation of purulent discharge. At 3 months, the patient remained asymptomatic, and satisfactory soft tissue healing had been achieved with progressive bone remodeling.

At the 6‐month follow‐up, complete healing of the intraoral mucosa was evident, and the cutaneous fistula had resolved entirely, leaving only minimal residual scarring. Clinical examination confirmed preservation of inferior alveolar nerve function.

### 3.3. Patient Perspective

The patient expressed relief that the underlying cause of the persistent fistula had been identified and successfully treated after 1 month of symptoms. He reported no pain, sensory disturbance, or limitation in daily activities and expressed satisfaction with the healing process and the minimal residual scar.

## 4. Discussion

### 4.1. Epidemiology and Classification

Odontomas are widely recognized as the most prevalent odontogenic tumors, representing a significant portion of all such lesions in the maxillofacial region [1]. Although compound odontomas are more frequently observed in the anterior maxilla, complex odontomas, like the one described in this case, show a predilection for the posterior mandible [7]. The profile of our patient, a 22‐year‐old male, is consistent with the peak incidence reported in the literature, which typically occurs during the second decade of life, with no significant gender predilection [1]. Although hamartomatous in nature, their potential for continued growth justifies their classification as tumors that require active surgical management.

### 4.2. Clinical Presentation

Most odontomas are asymptomatic and are discovered incidentally during investigation of delayed tooth eruption or impaction. Conversely, the present case exhibited an unusual clinical presentation characterized by chronic infection, intraoral bone exposure, and a persistent cutaneous fistula.

Odontogenic cutaneous fistulas remain a diagnostic challenge because they frequently mimic dermatological diseases, infected epidermoid cysts, chronic osteomyelitis, or cervicofacial actinomycosis. Consequently, patients often receive repeated courses of antibiotics or undergo unnecessary dermatological procedures before the odontogenic origin is recognized [6]. In our patient, the presence of a chronic purulent fistula at the mandibular angle associated with exposed bone strongly suggested an underlying odontogenic infection, emphasizing the importance of systematic intraoral examination in every patient presenting with a cervicofacial fistula [6, 8].

Although the final histopathological diagnosis was a complex odontoma associated with a dentigerous cyst, the potential contribution of the cyst to the chronic infection should also be considered. Dentigerous cysts associated with impacted teeth may become secondarily infected and can therefore contribute to chronic inflammatory or infectious manifestations. In this case, the chronic infection cannot be attributed exclusively to the complex odontoma, and the associated dentigerous cyst may have played a contributory role.

### 4.3. Diagnostic Assessment

Radiographically, the lesion displayed the classical appearance of a complex odontoma, consisting of a dense radiopaque mass surrounded by a thin radiolucent rim corresponding to the connective tissue capsule. The lesion caused marked displacement of the mandibular third molar toward the inferior border of the mandible, reflecting its progressive growth [4, 5].

Although panoramic radiography established the presumptive diagnosis, CBCT was indispensable for both diagnosis and treatment planning. Three‐dimensional evaluation accurately demonstrated the lesion′s dimensions, internal architecture, cortical thinning, and its intimate relationship with the inferior alveolar canal. These findings substantially influenced surgical decision‐making by enabling a minimally invasive approach while minimizing the risks of nerve injury and pathological fracture [4]. Similar observations have been reported by Assiri et al., who emphasized the value of CBCT in improving the diagnosis and surgical management of odontomas. [4]

The principal differential diagnoses included chronic osteomyelitis, cemento‐ossifying fibroma, osteoma and calcifying epithelial odontogenic tumor. However, the characteristic radiological appearance, association with an impacted third molar, and definitive histopathological findings established the diagnosis of a complex odontoma associated with a dentigerous cyst.

### 4.4. Therapeutic Considerations

This case posed several important therapeutic challenges. The lesion occupied the mandibular angle, an anatomically vulnerable region already weakened by cortical thinning and displacement of the impacted third molar. Conventional osteotomy using rotary burs could therefore have increased the risk of excessive bone removal, inferior alveolar nerve injury, and iatrogenic mandibular fracture. [9]

For these reasons, piezoelectric surgery was selected. Ultrasonic bone cutting offers greater precision, selective mineralized tissue removal, and preservation of adjacent soft tissues through micrometric oscillations. In addition, the cavitation effect improves visibility by reducing intraoperative bleeding. These advantages have been associated with lower postoperative morbidity and improved preservation of neurovascular structures compared with conventional rotary instruments [9, 10].

In the present case, complete enucleation of the odontoma, extraction of the displaced third molar, and debridement of the infected fistulous tract were successfully achieved without intraoperative complications or mandibular fracture.

### 4.5. Outcome and Prognosis

Histopathological examination confirmed the diagnosis of a complex odontoma associated with a dentigerous cyst. Complete surgical removal resulted in progressive resolution of both intraoral and extraoral lesions.

At the 6‐month follow‐up, complete healing of the oral mucosa and cutaneous fistula was observed, with preservation of inferior alveolar nerve function and no radiographic evidence of recurrence. These findings are consistent with previous reports demonstrating that complete excision of both the odontoma and its surrounding capsule is associated with an excellent long‐term prognosis and an extremely low recurrence rate [8, 9].

### 4.6. Strengths and Clinical Lessons

This case illustrates several clinically relevant points. First, chronic cutaneous fistulas of the cervicofacial region should always raise suspicion of an odontogenic origin, even in the absence of dental pain. Second, CBCT plays a fundamental role in evaluating large mandibular odontomas by accurately depicting their relationship with adjacent anatomical structures and guiding surgical planning. Finally, piezoelectric surgery represents a valuable alternative to conventional rotary instruments for the management of large mandibular odontomas because it enhances surgical precision while reducing the risks of nerve injury and pathological fracture.

The principal limitation of this report is the relatively short follow‐up period of 6 months. Although recurrence after complete excision is exceptionally rare, longer clinical and radiographic surveillance would further confirm the long‐term stability of the surgical outcome.

## 5. Conclusion

This case highlights the importance of considering an odontogenic origin in patients presenting with persistent cervicofacial cutaneous fistulas. Although complex odontomas are benign developmental lesions, secondary infection may lead to uncommon but clinically significant complications, including chronic fistula formation and bone exposure.

The combined use of panoramic radiography and CBCT allowed precise diagnosis and comprehensive preoperative assessment, whereas piezoelectric surgery enabled safe removal of the lesion with preservation of mandibular integrity and the inferior alveolar nerve.

Complete surgical excision remains the treatment of choice and provides an excellent prognosis. This case further supports the integration of three‐dimensional imaging and minimally invasive ultrasonic surgery in the management of large mandibular odontomas, particularly when anatomical complexity increases the risk of surgical complications.

## Author Contributions

**Imen Raâdani:** conceptualization, investigation, data curation, formal analysis, visualization, writing – original draft, writing – review and editing.

## Funding

No funding was received for this manuscript.

## Disclosure

The author has read and approved the final version of the manuscript. The corresponding author has full access to all of the data in this study and takes complete responsibility for the integrity of the data and the accuracy of the data analysis.

## Ethics Statement

Ethical approval was not required for this single anonymized case report according to our institutional policy. Written informed consent for publication of the clinical information and accompanying images was obtained from the patient.

## Conflicts of Interest

The author declares no conflicts of interest.

## Supporting information


**Supporting Information** Additional supporting information can be found online in the Supporting Information section. CARE checklist.

## Data Availability

Data sharing is not applicable to this article as no datasets were generated or analyzed during the current study.
