# Supplementary material for: Infected Complex Odontoma Associated With a Dentigerous Cyst Presenting as a Cutaneous Fistula in the Mandibular Angle: Surgical Management Using Piezoelectric Surgery
Source: Case Rep Dent. 2026 Oct 12;2026:2059316. doi: 10.1155/crid/2059316 (PMC13656789; doi:10.1155/crid/2059316)
Supplement: Supplementary file 1 [file CRID-2026-2059316-s001.docx]

**CARE  Checklist  of  information  to  include  when  writing  a  case  report**

**Topic	Item	Checklist item description	Reported on Line**

**Title	1**	The diagnosis or intervention of primary focus followed by the words “case report” . . . . . . . . . . . . . . . . . . . . . . . . . . yes

**Key Words	2**	2 to 5 key words that identify diagnoses or interventions in this case report, including "case report" . . . yes

**Abstract**

**(no references)**

**3a**	Introduction: What is unique about this case and what does it add to the scientific literature? . . . . . . . . . . . . . . . . yes

**3b**	Main symptoms and/or important clinical findings . . . . . . . . . . . . . . . . . . . . . . . . . . . . . . . . . . . . . . . . . . . . . . . . . . . . . . . yes

**3c**	The main diagnoses, therapeutic interventions, and outcomes . . . . . . . . . . . . . . . . . . . . . . . . . . . . . . . . . . . . . . . . . . . yes

**3d**	Conclusion—What is the main “take-away” lesson(s) from this case? . . . . . . . . . . . . . . . . . . . . . . . . . . . . . . . . . . . . . yes

**Introduction	4**	One or two paragraphs summarizing why this case is unique (**may include** reference**s**) . . . . . . . . . . . . yes

**Patient Information	5a**	De-identified patient specific information. . . . . . . . . . . . . . . . . . . . . . . . . . . . . . . . . . . . . . . . . . . . . . . . . . . . yes

**5b**	Primary concerns and symptoms of the patient. . . . . . . . . . . . . . . . . . . . . . . . . . . . . . . . . . . . . . . . . . . . . . . . . . . . . yes

**5c**	Medical, family, and psycho-social history including relevant genetic information . . . . . . . . . . . . . . . . . yes

**5d**	Relevant past interventions with outcomes . . . . . . . . . . . . . . . . . . . . . . . . . . . . . . . . . . . . . . . . . . . . . . . . . . . . . . . . yes

**Clinical Findings**

**Timeline**

**Diagnostic**

**Assessment**

**Therapeutic**

**Intervention**

**Follow-up and**

**Outcomes**

**6**	Describe significant physical examination (PE) and important clinical findings. . . . . . . . . . . . . . . . . . . . . . . yes

**7**	Historical and current information from this episode of care organized as a timeline . . . . . . . . . . . . . . . yes

**8a**	Diagnostic testing (such as PE, laboratory testing, imaging, surveys). . . . . . . . . . . . . . . . . . . . . . . . . . . . . . . yes

**8b**	Diagnostic challenges (such as access to testing, financial, or cultural) . . . . . . . . . . . . . . . . . . . . . . . . . . . . . yes

**8c**	Diagnosis (including other diagnoses considered) . . . . . . . . . . . . . . . . . . . . . . . . . . . . . . . . . . . . . . . . . . . . . . . . . yes

**8d**	Prognosis (such as staging in oncology) where applicable . . . . . . . . . . . . . . . . . . . . . . . . . . . . . . . . . . . . . . . . . yes

**9a**	Types of therapeutic intervention (such as pharmacologic, surgical, preventive, self-care) . . . . . . . . . . . . . . . . . . . . yes

**9b**	Administration of therapeutic intervention (such as dosage, strength, duration) . . . . . . . . . . . . . . . . . . . . . . . . . . . . . yes

**9c**	Changes in therapeutic intervention (with rationale) . . . . . . . . . . . . . . . . . . . . . . . . . . . . . . . . . . . . . . . . . . . . . . . . . . . . yes

**10a**	Clinician and patient-assessed outcomes (if available) . . . . . . . . . . . . . . . . . . . . . . .. . . . . . . . . . . . . . . . . . . . . . . . . . . . yes

**10b**	Important follow-up diagnostic and other test results . . . . . . . . . . . . . . . . . . . . . . . . . . . . . . . . . . . . . . . . . . . . . . . . . . . . yes

**10c**	Intervention adherence and tolerability (How was this assessed?) . . . . . . . . . . . . . . . . . . . . . . . . . . . . . . . . . . . . . . . . . yes

**10d**	Adverse and unanticipated events . . . . . . . . . . . . . . . . . . . . . . . . . . . . . . . . . . . . . . . . . . . . . . . . . . . . . . . . . . . . . . . . . . . yes

**Discussion	11a**	A scientific discussion of the strengths AND limitations associated with this case report . . . . . . . . . . . . . . . . . . . . . . . yes

**11b**	Discussion of the relevant medical literature **with references**. . . . . . . . . . . . . . . . . . . . . . . . . . . . . . . . . . . . . . . . . . yes

**11c**	The scientific rationale for any conclusions (including assessment of possible causes) . . . . . . . . . . . . . . . . . . . . . . . . yes

**11d**	The primary “take-away” lessons of this case report (without references) in a one paragraph conclusion . . . . . . . yes	**Patient Perspective	12**	The patient should share their perspective in one to two paragraphs on the treatment(s) they received . . . . . . . . yes	**Informed Consent	13**	Did the patient give informed consent? Please provide if requested . . . . . . . . . . . . . . . . . . . . . . . . . . . . . . . . . . . . . . **Yes *	No**
